# Integrated care in Germany—a stony but necessary road!

**DOI:** 10.5334/ijic.853

**Published:** 2012-03-27

**Authors:** Volker Amelung, Helmut Hildebrandt, Sascha Wolf

**Affiliations:** Professor for International Healthcare Systems Research, Medical University of Hannover, President, German Managed Care Association (BMC), Bundesverband Managed Care e.V., Friedrichstraße 136, 10117 Berlin, Germany; CEO, OptiMedis AG, Borsteler Chaussee 53, 22453 Hamburg, Germany; Managing Director, German Managed Care Association (BMC), Friedrichstraße 136, 10117 Berlin, Germany

**Keywords:** integrated care, German healthcare system, provision of care in Germany

## Abstract

German healthcare provides a very comprehensive benefits catalogue, high quality standards, low access barriers and in particular healthcare which is independent from one’s income. But at the same time it is one of the most expensive systems in the world. Reasons for the high costs of care are mainly due to the separation of the outpatient, inpatient and rehabilitation sectors, the poor information flow between the service providers and insufficient competition in healthcare provision. In the last 15 years the German government has introduced various reform acts and in doing so has followed a continual path of development: more competition for care concepts between health insurances, more options for the insured and more leeway for players in the various sectors of healthcare. The following article gives an overview of new forms of contracting that have been introduced and provides recommendations for the further development of integrated care in the German healthcare system.

## Introduction

During the last 15 years, the German government has introduced various reform acts to improve quality and efficiency of medical provision. In this paper we describe the development of integrated care, new forms of contracting that have been introduced, and provide political recommendations.

German healthcare provides a very comprehensive benefits catalogue, high quality standards, low access barriers and in particular healthcare which is independent from one’s income for the entire population. However, on the basis of their gross domestic product, this also places Germany among the most expensive healthcare systems worlwide. In addition, great challenges are to come. In the context of the demographic developments and progress in medical technology, the number of patients with chronic diseases is likely to increase remarkably. Thus the quality and economic efficiency of a healthcare system will always depend on how the care of people with complex disease patterns is organised. This is where German healthcare reaches its limits, as it makes few allowances for customised treatment processes. This is particularly due to the extensive separation of the outpatient, inpatient and rehabilitation sectors, the poor information flow between the service providers and the insufficient competition in healthcare provision. It is even more regrettable that the German government’s many attempts to provide integrated care have largely been unsuccessful and their spread has faltered since its start-up funding expired at the end of 2008. Thus there is a broad consensus that the German healthcare system must undergo structural reforms.

## Where is the problem in German healthcare?

The remaining inefficiency of German healthcare is not a new issue. Already in the 1970s the inadequate exchange of information between doctors was criticised as well as the ruptures in treatment when transitioning from one medical sector to another [1]. Since then little has changed, as the current comprehensive study which compares 11 countries has confirmed [2]. Twenty-three percent of the patients surveyed in Germany affirmed that the flow of information between the service providers is insufficient. That is more than in any other country in the study. Thirty-five percent complained that specialists are not sufficiently familiar with the disease history of the patient or that the primary care providers are not informed about the treatments by specialists. These informational deficiencies directly lead to the accrual of preventable costs: according to the Bertelsmann Stiftung health monitor, every fourth person surveyed believed that the interface problems in transferring from the primary care to specialist treatment resulted in unnecessary repeat examinations. In the case of transfers from outpatient to inpatient care, as much as 27.5% are of this opinion [3].

The great lack of the integration of technical processes and informational technology is the consequence of three fundamental problems in German healthcare. First, the competition for contracts and the provision of care is still very limited. Instead care is dominated by mutual and uniform contracts between the traditional associations of the statutory sickness funds and the service providers. There is too little room in this system for individualised, patient-orientated services. The lack of competition all too often leads to reliance on the familiar and thus results in a certain aversion to technology. Secondly, there is a lack of cooperation between disciplines. The field of activities of German physicians (in comparison to other countries) is characterised by its wide spectrum and tasks that more affordable and possibly even more suitable professions could execute just as well. Thirdly, medical care takes place in largely separate sectors with their own compensation systems, budgets, high market entry barriers and complex planning structures. A lack of transparency and false incentives lead to the fact that the diagnostic process is continually further developed without considering previous test results. This is not only a problem at the interface between outpatient and inpatient treatment, but also within sectors, such as the decision process between primary care physicians and specialists [4].

## Integrated care—ambiguous development since the end of start-up financing

In the last 15 years the German government has introduced various laws and in doing so has followed a continual path of development: more competition for care concepts between health insurances, more options for the insured and more leeway for players in the various sectors of healthcare [5]. In the meantime, numerous different possibilities for contracts with managed care elements in particular forms of care have been created (Table 1).

The German government has placed its hopes in managed care to a large extent. Since 2004 individual physicians and physician networks have been able to become direct contract partners with the health insurances for the first time through selective contracts. Combined with an attractive start-up financing of up to 1% of the entire compensation of physicians and hospitals, ca. €680 million, integrated care experienced a strong push. Within four years, approximately 6000 contracts were concluded.

With the expiration of the start-up financing at the end of 2008, the success ebbed somewhat. Around 20% of the contracts were immediately dissolved or not renewed. At the end of 2009 it is assumed that there were approximately 5000 contracts and somewhat below 1% of the total expenses [7]. Although the sickness funds have drastically changed and acquired valuable expertise, the temporary start-up financing apparently did not suffice to fully attain the impact which was hoped for. It is even more regrettable since regional full-care models have proved to be able to improve the health and reduce the costs of sickness funds [8].

Today, due to the conclusion of a series of new contracts in the mental illness field, rheumatology and also in full-care models, we estimate approximately 6000 contracts and an overall share of perhaps 1.5% of the total expenses.

The healthcare reform which took effect on January 1st, 2012, the Versorgungsstrukturgesetz (care structure law), includes several good methods for interdisciplinary and cross-sector models of care. For example hospitals’ obligation to mandatory discharge management. The introduction of an outpatient specialist care sector, in which inpatient as well as SHI-authorized physicians can equally take part, can also contribute to lessening the frictional losses at sector boundaries. The change to § 87b SGB V is particularly important. It creates the opportunity to introduce separate compensation rules for network practices and to assign these their own fee volumes. In this way quality- and success-based forms of compensation (pay for performance) could receive new vigour. However, beyond these specific improvements the central blockades for integrated care are not addressed by the care structure law.

## What must be done?

If one goes looking for blockades in integrated care, one usually encounters the supposed inflexibility of traditional structures, the conflicts between statutory doctors and those working in inpatient care, as well as the fears of losing independence and worries about the transparency of quality shortcomings [9]. These problems, which are to a certain extent more feared than actual, surely play a role in explaining the great caution with which players in healthcare approach new contract forms. In the end, however, this only concerns the symptoms of inadequate conditions which insufficiently take into account the improvement of outcomes. For the belief has become established that traditional structures must be broken up due to the expected increase in chronic and complex illnesses. Policies, sickness funds, service providers, consultants and scientists confirm the need for more cross-sector and interdisciplinary coordination in order to guarantee holistic treatment processes without breaks in care as well as to prevent unnecessary multiple examinations. It is even more remarkable that up until now no dynamic could develop in the direction of innovative forms of care. The sobering inertia of those involved has many causes. In the end, one thing is clear: integrated care will only become established when all those involved see a realistic chance for personal added value through managed care.

Currently the risks outweigh the advantages for the health insurance companies, since innovative forms of care usually require considerable investments. However, as a corporation under public law a statutory sickness fund is obligated to demand additional premiums from their policyholders when the insurance exceeds its budget. Experience with additional premiums has shown that these can occasionally have serious consequences in terms of price competition. The risk and the use of resources necessary for integrated care are thus often classified as unreasonably high. Instead it is often more attractive for health insurances to invest in comprehensive, widespread primary care physician contracts or in contracts which are not cross-sector and thus easier to manage. In order to change this, health insurances should be given more flexible entrepreneurial leeway which also allows them to invest in projects whose revenue may first accrue in three to five years. In addition, innovation budgets should be introduced in health insurances for the development of highly innovative forms of care and pilot projects. The appropriate use of funds can be determined by using standardised evaluations of the supported projects.

There is also too little initiative from the service providers, as the economic pressure to be involved in selective contracts is rather low [10]. Although many are dissatisfied with the high work load and the earning potential in statutory healthcare, none of those involved have to fear being excluded from the system. On the other hand, many physicians fear that a change in the structure of care could restrict their authority, increase surveillance and interfere with their freedom of treatment. For this reason, beyond the compensation system, incentives should be offered to physicians for their participation in integrated care. Compensation based on pay for performance would enable each party to attain additional income through individual performance. At the same time, this would enforce the desired quality-based competition. By partly transferring the morbidity risk to the physician and by profit sharing, volume increases can be effectively prevented. In addition, special compensation rules for physician networks should be instituted and they should be assigned their own compensation budget.

The insured are rather skeptical of new forms of care. Most of them are confident in the quality supposedly provided by the government in the standard provision of care. Since the advantages of innovative forms of care can first be recognised when they are made use of in cases of illness, the insured are not very willing to accept alternative forms of care which are designed for long-term use. Thus it is even more important to ensure more transparency. Currently the insured can hardly judge the different services of the health insurances due to a lack of information. Thus more standardised evaluations and the publication of results are absolutely necessary. With the rising level of transparency, new forms of care would become more significant as an instrument for competition for the health insurances and a dynamic development process would be set in motion.

Comparable to the reluctance of the other participants, private partners—whether private insurances, the industry or also private financers, such as banks and private equity—have been rather cautious about investing in forms of integrated care up until now. There are individual cases of involvement, but the scale of engagement is nowhere close to the requirement for modernisation and increasing efficiency in German healthcare.

## Conclusions

In the future the efficiency of a healthcare system will be particularly assessed based on how the treatment of people with complex disease patterns is organised. The German healthcare system is not yet sufficiently equipped for this challenge. The far-reaching separation of the outpatient and inpatient sector, the inadequate flow of information between the service providers and competition in contract and care provision, which is still too weak, all allow too little room for customised treatment processes which are optimised throughout all of the stages of care. Thus it is all the more important to finally enable the breakthrough of cross-sector and interdisciplinary forms of care. For it is only through cooperation and holistic treatment workflows that unnecessary repeat examinations can be prevented, individual, custom therapies as well as an efficient form of innovation-based competition can be made possible. Selective contracts will only become established when three prerequisites are fulfilled: first, the policyholders must be convinced that the quality of the products offered by the health insurances differs from insurance to insurance. Secondly, they must be able to recognise these differences and to assess the advantages and disadvantages for themselves. Thirdly, the service providers, statutory health insurances and possibly third-party financers have to see a realistic chance to earn profits through the participation in innovative forms of care.

Overcoming barriers in integrated care seems to be a never-ending story in the German healthcare system. Although politics have introduced more leeway for medical providers and insurances through various reform acts, the competition for contracts and the provision of care is still very limited. The separation of outpatient, inpatient, and rehabilitation sectors is stricter than in other countries. Nevertheless, we feel confident that we are on the right path in the direction of a more patient-oriented system. In the last decades, the number of physician networks has doubled from 200 to 400. By now, 30,000 doctors participate in cooperative forms of medical treatment. This builds the foundation for numerous population- and indication-based concepts of integrated care which have made remarkable progress. Yet, there is still a lot of work to do. At the end, integrated care will only become established when all stakeholders see a realistic chance to earn medical and economical profits through managed care.

## About the authors

**Prof. Dr. Volker E. Amelung** is professor for international healthcare systems Research at the Medical University of Hannover, Germany. He is also the president of the German Managed Care Association (BMC), Berlin. His research activities concentrate on healthcare policy, managed care, healthcare management, and healthcare systems research. He has co-authored more than ten books, and has published over 100 papers on managed care, healthcare policy, new technologies, and integrated delivery systems.

**Helmut Hildebrandt** is CEO and owner of OptiMedis AG, located in Hamburg, Germany. He holds a pharmacy diploma from Philipps-University, Marburg, Germany. He has worked intensively with the World Health Organization (Copenhagen) and with the institute for medicine-sociology of the University of Hamburg. Being President and CEO of a successful consulting firm for twenty years, he cooperated with a wide array of organizations in healthcare industry, such as leading hospitals, sickness funds, health insurances, physician networks, pharmaceutical and biomedical companies, ministries, and others. Mr. Hildebrandt is board member of the BMC.

**Dr. Sascha Wolf** is Managing Director of the BMC. The BMC is one of the leading independent healthcare associations, elaborating innovative concepts in healthcare management and healthcare policy. It affiliates approximately 160 members from different areas in healthcare, such as leading sickness funds, health insurances, consultancy firms, pharmaceutical companies, biomedical companies, and others. Prior to this he was responsible for health politics at the Economic Council to the Christian Democratic Union of Germany (CDU).

## Figures and Tables

**Table 1.**
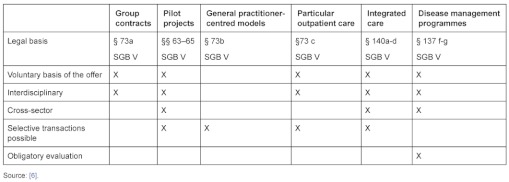
Special forms of the provision of healthcare in Germany.
